# Low-Temperature Toluene Oxidation on Fe-Containing Modified SBA-15 Materials

**DOI:** 10.3390/molecules28010204

**Published:** 2022-12-26

**Authors:** Ivalina Trendafilova, Manuel Ojeda, John M. Andresen, Alenka Ristić, Momtchil Dimitrov, Nataša Novak Tušar, Genoveva Atanasova, Margarita Popova

**Affiliations:** 1 Institute of Organic Chemistry with Centre of Phytochemistry, Bulgarian Academy of Sciences, Acad. G. Bonchev Str., Bl. 9, 1113 Sofia, Bulgaria; 2 Laboratory of Inorganic Materials Chemistry, Namur Institute of Structured Matter, University of Namur, 5000 Namur, Belgium; 3 Research Centre for Carbon Solutions (RCCS), Heriot-Watt University, Edinburgh EH14 4AS, UK; 4 Department of Inorganic Chemistry and Technology, National Institute of Chemistry, Hajdrihova 19, 1000 Ljubljana, Slovenia; 5 Graduate School, University of Nova Gorica, Vipavska 13, 5000 Nova Gorica, Slovenia; 6 Institute of General and Inorganic Chemistry, Bulgarian Academy of Sciences, Acad. G. Bonchev Str., Bl. 11, 1113 Sofia, Bulgaria

**Keywords:** iron oxides, AlSBA-15, ZrSBA-15, SBA-15, toluene oxidation, VOC removal

## Abstract

Transition metals as catalysts for total VOC oxidation at low temperatures (150–280 °C) are a big challenge nowadays. Therefore, iron-modified SBA-15, AlSBA-15, and ZrSBA-15 materials with 0.5 to 5.0 wt.% Fe loading were prepared and tested for toluene oxidation. It was found that increasing Fe loading significantly improved the rate of oxidation and lowered the temperature of achieving 100% removal of toluene from above 500 °C for the supports (AlSBA-15 and ZrSBA-15) to below 400 °C for 5FeZrSBA-15. The formation of finely dispersed iron oxide active sites with a particle size less than 5 nm was observed on all the SBA-15, AlSBA-15, and ZrSBA-15 supports. It was found that the surface properties of the mesoporous support due to the addition of Al or Zr predetermined the type of formed iron oxide species and their localization on the support surface. Fe-containing SBA-15 and AlSBA-15 showed activity in total toluene oxidation at higher temperatures (280–450 °C). However, 5 wt. % Fe-containing ZrSBA-15 showed excellent activity in the total oxidation of toluene as a model VOC at lower temperatures (150–380 °C) due to the synergistic effect of Fe-Zr and the presence of accessible and stable Fe^2+^/Fe^3+^ active sites.

## 1. Introduction

There are increasing environmental concerns regarding anthropogenic releases of volatile organic compounds (VOCs) that have a serious impact on the environment since they are linked to ozone formation, acid rain, and the formation of fine particles and smog. [1,2]. Another reason for VOCs’ strict monitoring, regulation and mandated reduction is their effect on human health, where some VOCs are suspected or proven carcinogens, linked to respiratory illnesses and may interfere with vital body functions [3,4,5]. A promising approach for VOCs reduction has been the application of catalytic oxidation [6,7,8]. The main challenge to achieve high efficiency of this process is the development of a stable, cost-effective catalytic system with high conversion of VOCs into non-pollutants. Widely used oxidation catalysts with superior activity are noble metals (Pt, Pd, Au, Ag, Ru) that may have prohibited cost issues for many applications. Hence, there is a great focus by many industrial and academic research groups to identify non-noble metal catalysts, such as iron supported on porous media including SBA-15 [9,10,11].

Mesoporous silica materials have been touted as suitable supports in heterogeneous catalysis for oxidation reactions, because of their unique properties [12]. They have high specific surface areas that provide easy access for VOC molecules to reach the active sites. This is linked with their uniform channel structure and tunable pore size that allow for even dispersion of the catalytic species throughout the whole volume of the material. In addition, they have high mechanical and chemical stability that allows long-term usage. SBA-15 is one of the most used mesoporous silica supports, due to its pore-size tunability [13].

Materials with proven catalytic activity for the combustion of VOCs at lower temperatures (150-280 °C) are noble metals [9,10,11], but they suffer from major drawbacks such as price, availability, geographical distribution, and tendency to self-poisoning. A suitable alternative to the noble metals in reactions of total oxidation of VOC was given by supported oxides of transition metals (Fe, Ti, V, Cr, Cu, Mn, etc.) [14,15]. However, they usually work at high temperatures (280–450 °C). The properties of the loaded metal oxide species (oxidation state, size, and dispersion) can be strongly influenced by the applied synthesis procedure, the metal precursor, the incorporation of the catalytic active oxide onto the support, and the type of support materials [16,17,18,19,20,21,22,23,24,25,26]. Along with its good catalytic activity, iron is readily available at a low cost and environmentally friendly, which makes it an attractive candidate as the active phase in catalysts with industrial application.

In the present work, Fe-containing SBA-15, ZrSBA-15, and AlSBA-15 were synthesized and tested for the removal of toluene as a model compound for the oxidation of VOCs. Toluene was chosen as it represents the major class of aromatic pollutants in harmful emissions, and it is considered an important compound with photochemical ozone creativity potential and a hazardous air pollutant with serious negative effects on human health [21,22]. The influence of the synthesis procedure and the chemical composition on the physicochemical and catalytic properties of the obtained materials was studied.

## 2. Results and Discussion

### 2.1. Characterization

**Figure 1:**
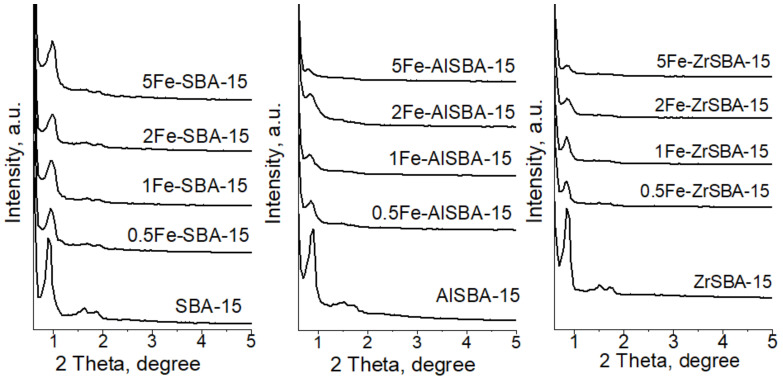
Low-angle XRD patterns of the studied samples.

Low-angle XRD patterns of SBA-15, AlSBA-15, and ZrSBA-15 samples show the presence of reflections at around 0.92, 1.64 and 1.85 2 Theta, which are typical for SBA-15 materials and correspond to (100), (110) and (220) hkl planes of ordered mesoporous structure with P6mm space group (Figure 1). A decrease in the structure ordering of AlSBA-15 and ZrSBA-15 with the increase in the iron content is observed. The partial collapse of the ordered structure is evident for the samples with the highest amount of iron. XRD patterns at higher diffraction angles of all metal-containing samples show no reflections characteristic of metal/metal oxide phases and only two diffraction peaks due to the sample holder (Figure SD1), indicating that all metal/metal oxide nanoparticles present in these samples are highly dispersed and smaller than 5 nm.

**Figure 2:**
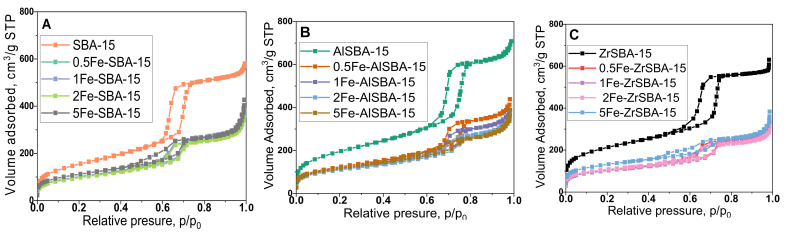

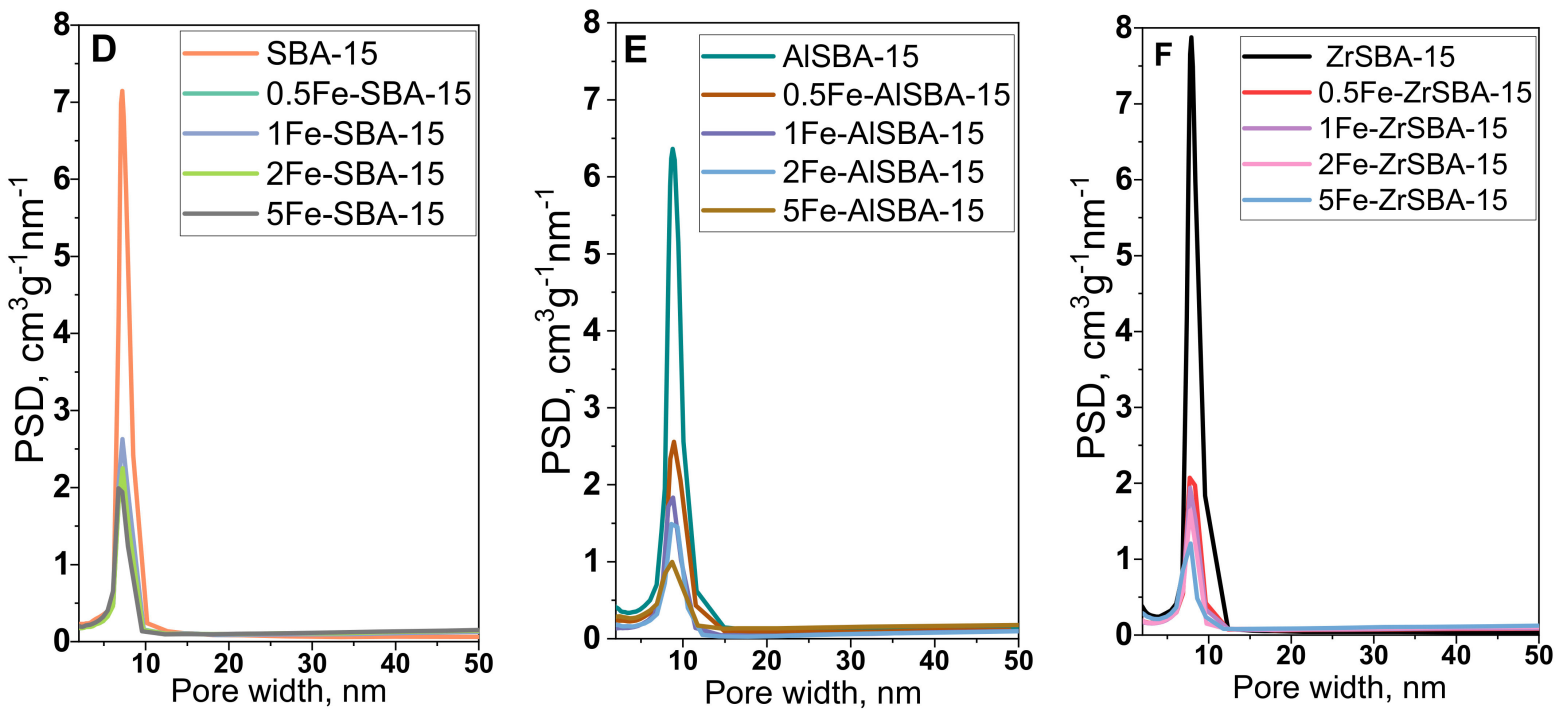
Nitrogen physisorption isotherms (**A**–**C**) and pore size distributions (**D**–**F**) of the samples. (**A**,**D**) Fe-modified SBA-15; (**B**,**E**) Fe-modified AlSBA-15; (**C**,**F**) Fe-modified ZrSBA-15.

The nitrogen sorption isotherms for SBA-15 are typical type-IV, characteristic of ordered mesoporous silicas with 2D-hexagonal pore arrangement [27], while modifications with aluminum and zirconium during direct synthesis caused some mesopore narrowing due to the presence of metal oxide nanoparticles (Figure 2A–C). Post-synthesis modification with iron into SBA-15, AlSBA-15, and ZrSBA-15 also led to changes in the shape of the hysteresis loops. The hysteresis loops of all samples containing iron are closing down at lower relative pressure values in comparison with the original support, which indicates that the pores are partially narrowed with iron oxide nanoparticles. The increase in the amount of the deposited iron oxide nanoparticles into all supports resulted in an appreciable widening and tailing of hysteresis loops and in a two-step desorption [28], observed in the samples with the highest content of iron.

Pore size distributions of the different mesoporous materials have been determined using the BJH model widely used for this type of sample [29]. Although this model often underestimates pore sizes [30], it is appropriate for comparative purposes. The pore size distributions were determined from adsorption isotherms. As it can be observed, the maximum characteristics to open mesopores of SBA-15, AlSBA-15, and ZrSBA-15 are the most intense and show an average pore diameter of 7.1, 8.7, and 7.9 nm, respectively, indicating the increase of the average pore size due to introduction of Al and Zr during synthesis (Figure 2D–F). The less intensive maximums are characteristic of narrowed pores due to iron oxide deposition and a shift to lower pore size value in the samples with the highest iron content is observed as well.

The parameters calculated from the adsorption isotherms of the samples are summarized in Table 1. As it can be seen, the impregnation procedure leads to a decrease in the specific surface area with 35 to 40% for pure SBA-015 and AlSBA-15 samples, whereas for the ZrSBA-15 the decrease is higher, up to 50%. The ball milling modification procedure has a stronger effect than the decomposition of the salts on the surface of the mesoporous samples, leading to a decrease in the surface area. However, the increase in iron content has a positive effect on the preservation of the surface area during the applied modification procedure. The mesoporous structure of SBA-15 presented pores with a size range from 7 to 9 nm, which confirms that pore blockage is not evidenced.

Elemental analysis of the studied catalysts performed by EDX confirmed the successful incorporation of Al and Zr for all samples. Besides, the amount of iron loaded by grinding in the SBA-15, AlSBA-15, and ZrSBA-16 samples well corresponds to the theoretical iron contents.

**Figure 3:**
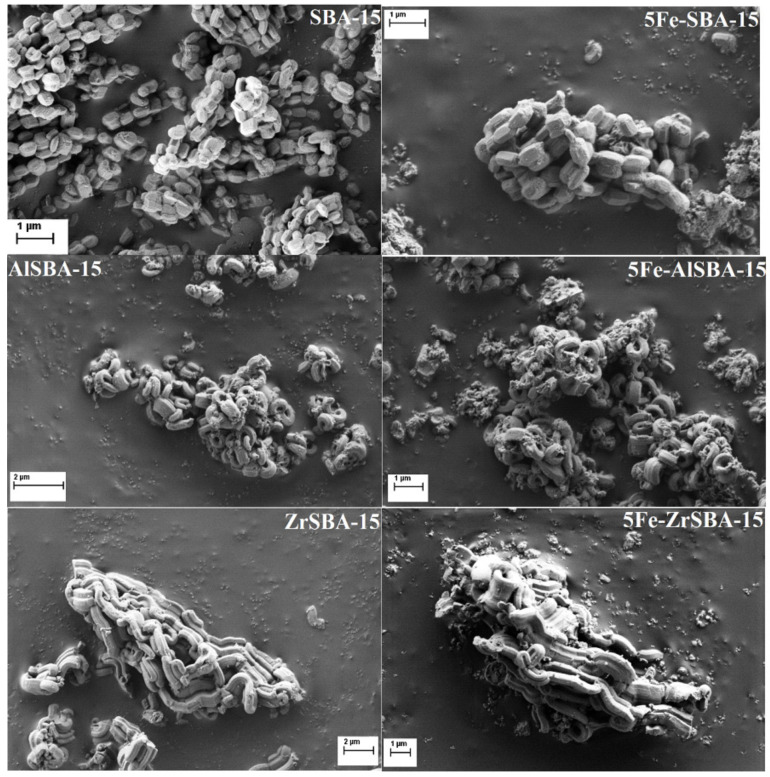
SEM images of the parent SBA-15, AlSBA-15, and ZrSBA-15 and their Fe modifications.

SEM images (Figure 3) confirmed the formation of hexagonal SBA-15 particles with sizes around 500 nm and narrow size distribution. It was observed that the post-synthesis procedure of Fe loading by grinding into the parent SBA-15 does not lead to disruption of the morphology of the support particles, no matter the % of loaded Fe (Figure 3). Preservation of the particle morphology means that the observed decrease of specific surface area and pore volume is a result of pore filling with iron species, and the grinding procedure. The addition of Al and Zr precursors during the formation of SBA-15 changed the shape of the particles for both samples. In the images presented in Figure 3, can be seen that the growth of the particles is more limited in two dimensions, but the growth in the third dimension does not stop as fast as for the SBA-15 material. This way the obtained particles can be said that are 2D nanomaterials, as this effect is more pronounced for the Zr-containing sample.

**Figure 4:**
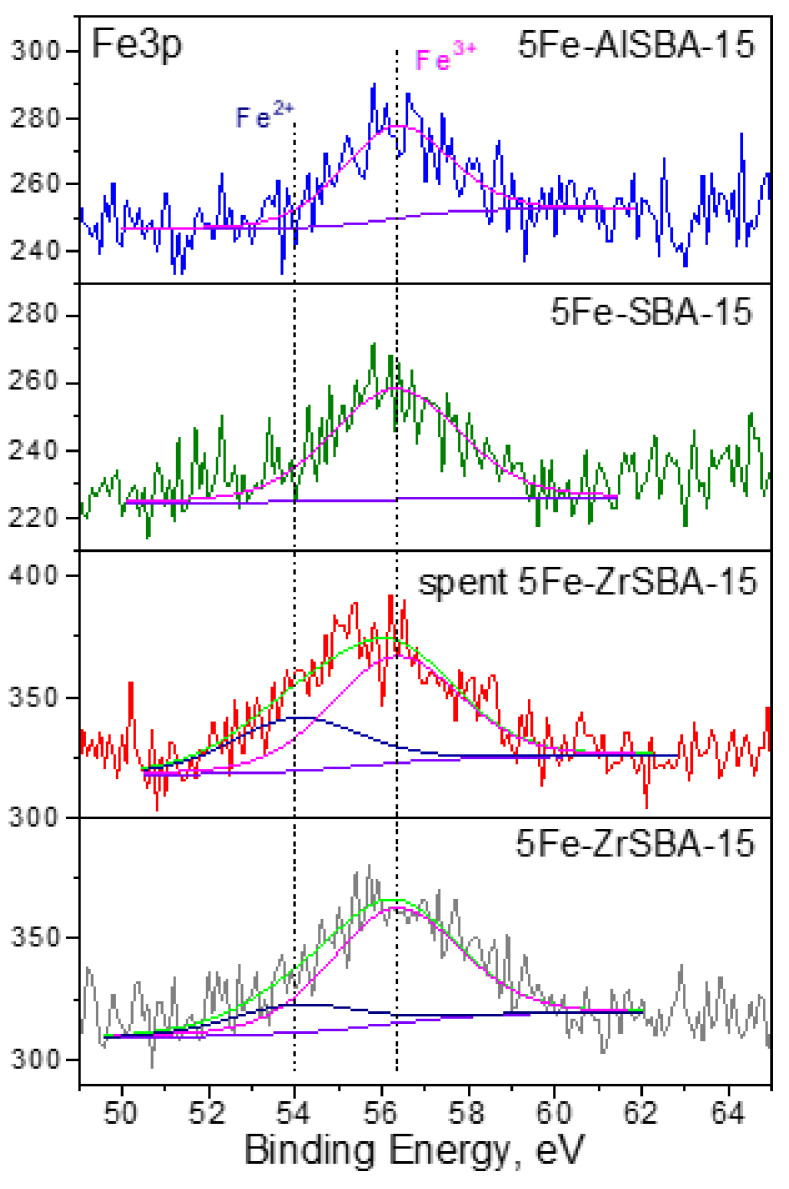
Fe3p XPS regions of the studied samples.

The surface chemical compositions of the Fe-modified samples with the highest content were additionally explored by XPS. The Fe 3p peak at 55.6 eV due to the presence of Fe^3+^ was detected for the 5Fe-SBA-15 and 5Fe-AlSBA-15 samples (Figure 4). The registered two Fe 3p peaks at 55.6 eV and at 53.7 eV in the spectrum of 5Fe-ZrSBA-15 could be ascribed to the presence of Fe^3+^ and Fe^2+^, respectively [31]. Based on the ratio of Fe^2+^/Fe^3+^, we could assume the predominant formation of Fe_3_O_4_ nanoparticles and a small part of Fe_2_O_3_. It is observed that the FWHM of Fe 3p for Fe^3+^ is larger than for Fe^2+^. Moreover, higher Fe content was detected for the 5Fe-ZrSBA-15 sample (0.8 at. %) in comparison with the 5Fe-AlSBA-15 (0.3 at. %) and 5Fe-SBA-15 (0.4 at. %) samples (Table 2). We could conclude that a higher content of Fe existing as finely dispersed Fe_3_O_4_ particles or as Fe^2+^/Fe^3+^ ions is formed on the surface of 5Fe-ZrSBA-15 (not detectable by XRD) [18].

**Figure 5:**
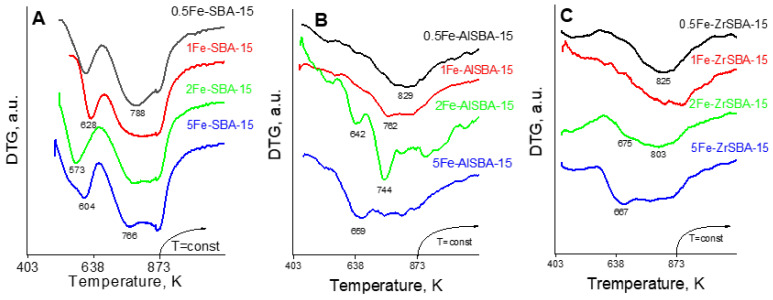
TPR-DTG profiles of the studied samples. (**A**) Fe-modified SBA-15; (**B**) Fe-modified AlSBA-15; (**C**) Fe-modified ZrSBA-15.

TPR-TG profiles of the metal-modified materials are shown in Figure 5. TPR results reveal the formation of iron oxide species with different reducibility depending both on the iron content and the support used. In the case of pure SBA-15 support, two well-defined effects are observed due to step-wise reduction that could be ascribed to the reduction of hematite to magnetite in the 500–670 K temperature range followed by their further reduction to metallic iron with temperature increase up to 873 K (Figure 5A) [32]. Besides, a well-visible tendency of a shift to low-temperature reduction is found with an increase in the iron content in the samples (see Table 1). More complicated is the status quo when iron is loaded on AlSBA-15 and ZrSBA-15. Here, no well-defined reduction effects are registered that could be assigned to the influence of the additional presence of Al and Zr within the samples on the state and reducibility of the loaded iron oxide species (Figure 5B,C). However, the most facilitated iron oxide reduction (up to 700 K) is found for the modifications with the highest iron loadings (5AlSBA-15 and 5ZrSBA-15) that could be ascribed to the presence of predominantly very finely dispersed iron oxide particles as it was evidenced by XPS.

### 2.2. Toluene Oxidation Catalytic Tests

**Figure 6:**
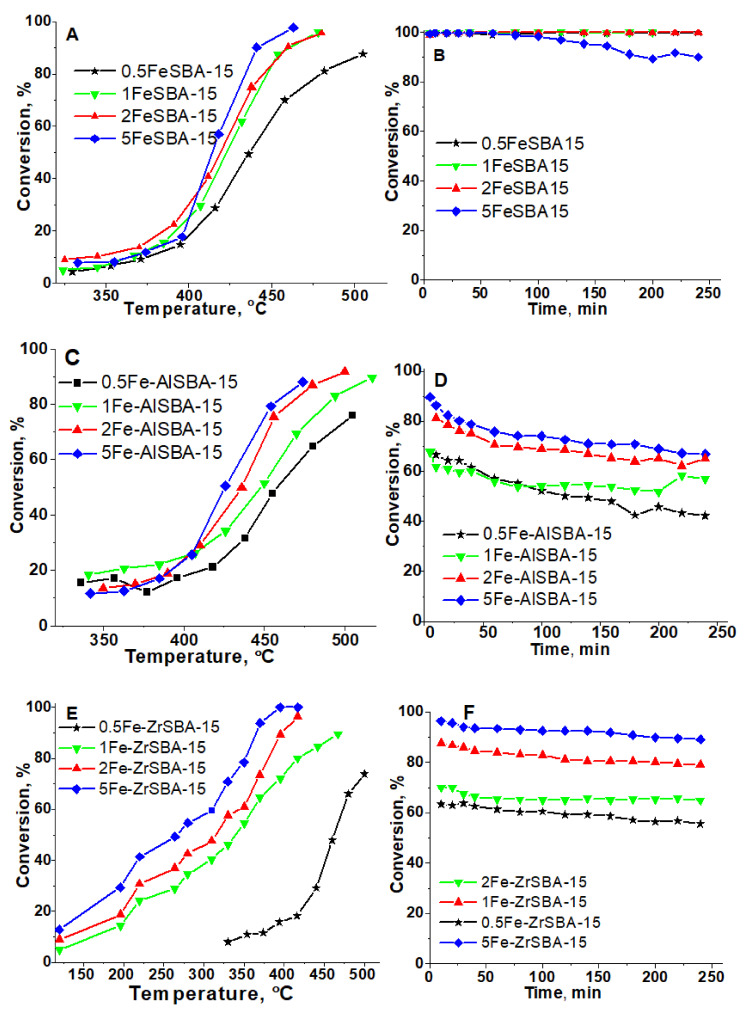
Toluene conversion vs. reaction temperature (**A**,**C**,**E**) and vs. time on stream (**B**,**D**,**F**) on the studied samples.

In Figure 6, the temperature and time on stream dependences of catalytic activity in the total oxidation of toluene on various iron-modified SBA-15, AlSBA-15, and ZrSBA-15 materials are shown. CO_2_ is the only registered carbon-containing product in all cases. Prior to the catalytic tests, the catalysts were pretreated in nitrogen at 673 K for 1 h in order to ensure the removal of moisture and physisorbed gases. The shift of the conversion curves of Fe-containing SBA-15, AlSBA-15, and ZrSBA-15 materials to lower temperatures with the increase in the iron content was detected. For the samples with the highest Fe-loading in both SBA-15 and AlSBA-15 materials, 100% of toluene conversion is reached at about 450 °C, while for the 5 wt. % Zr-modified SBA-15 total conversion is reached at the much lower reaction temperature of 380 °C. The highest activity of 5Fe-ZrSBA-15 could be explained by the higher content of Fe on the ZrSBA-15 surface and the presence of finely dispersed Fe_3_O_4_ particles or Fe^2+^/Fe^3+^ ions, which can be reduced at a lower temperature. The redox properties of the samples are very important considering the Mars–van Krevelen mechanism, which is widely accepted in the case of toluene oxidation reaction [33,34].

The stability of the catalytic performance for all samples was studied at 450 °C for 4 h (Figure 6B,D,F). The Fe-containing SBA-15 samples show stable catalytic activity, except 5Fe-SBA-15 where the conversion slightly decreases after 100 min TOS. Besides all Fe-containing AlSBA-15 catalysts show a constant decrease in activity in the TOS experiment (Figure 6D). The lowest content of accessible Fe^2+^/Fe^3+^ redox active sites in Fe-AlSBA-15, as shown by XPS data (0.3 at. %, Table 2), can be the reason for the observed deactivation trend. There are no changes in their physicochemical characteristics (XRD and XPS data) when fresh and spent catalysts are compared. At the same time, stable catalytic activity with time on stream was registered for all ZrSBA-15-modified samples (Figure 6F). The presence of finely dispersed Fe_3_O_4_ particles and/or Fe^2+^/Fe^3+^ ions could be a reason for the stable catalytic activity of 5Fe-ZrSBA-15. Additionally, the characterization of the fresh and spent 5Fe-ZrSBA-15 by XPS was performed in order to compare the composition and the state of metals in the catalyst before and after the catalytic test (Figure 4 and Figure SD2). A negligible decrease in the content of Fe and Zr was registered after the catalytic reaction (Figure SD2). A small increase in the Fe^2+^ is observed in the spent 5Fe-ZrSBA-15 catalyst in comparison with the fresh one as well. Zirconium 3d spectral regions of fresh and spent 5Fe-ZrSBA-15 can be fitted with two pairs of Zr 3d_5/2_ and Zr 3d_3/2_ peaks with a characteristic spin–orbital splitting of 2.4 eV (Figure SD2). In the case of the fresh 5Fe-ZrSBA-15, the lower Zr 3d_5/2_ binding energy at 182.3 eV is in the range of Zr(IV) in ZrO_2_, whereas the higher energy component at 183.6 eV corresponds to the formation of Zr(IV) species bound to more electron attractive species [35]. The higher intensity of the Zr 3d_5/2_ peak detected for 5Fe-ZrSBA-15 shows the predominant crystalline ZrO_2_ formation. There are no changes in the state of Zr after the catalytic test. We can assume that the stability of the ZrSBA-15 support predetermines the stability of the supported Fe oxide species. Moreover, the 5Fe-ZrSBA-15 catalyst was studied in isothermal conditions (4 h at 450 °C reaction temperature) in 6 reaction cycles. The significant changes in the activity and physicochemical properties of the catalyst are not observed, the activity at the end of the first reaction cycle was 89.4 and at the end of the sixth reaction cycle was 88.5%.

## 3. Experimental

### 3.1. Synthesis of the Catalysts

Pure SBA-15 mesoporous silica material was obtained as follows: triblock copolymer Pluronic P123 (PEO-PPO-PEO) used as a surfactant (8.0 g) was dissolved in deionized water (260 mL) and HCl (12M, 40 mL) by continuous stirring, and the solution was kept at 40 °C for 2 h. On complete dissolution, TEOS (7.0 g) was added dropwise to the above solution. The mixture was then left stirring for 24 h at 40 °C and subsequently subjected to hydrothermal treatment at 100 °C for 48 h in an oven. The white solid formed was filtered off and dried at 60 °C. The template was removed by calcination at 600°C for 8 h [23].

For the samples containing Al or Zr, their precursors were introduced during the synthesis of the SBA-15 together with TEOS, and the reaction was conducted at a pH of 1.5. Al Isopropoxide and ZrONO_3_·xH_2_O (Sigma-Aldrich, Germany) were used as Al and Zr sources, respectively. The theoretical ratios of Si/Al = 20 and Si/Zr = 10 were applied.

Functionalization of the parent SBA-15, AlSBA-15, and ZrSBA-15 supports with iron species was achieved by post-synthesized approach grinding the silica with the (Fe(NO_3_)_3_·9H_2_O), to reach loadings from 0.5 to 5.0 wt% in a Retsch PM-100 planetary ball mill using a 125 mL reaction chamber and eighteen 10 mm stainless steel balls. Optimized milling conditions were 10 min at 350 rpm as previously reported in the literature [12,24]. The material was then sintered at 600 °C for 4h under air using a heating rate of 10 °C·min^−1^. The obtained samples were named xFeSBA-15, xFe-AlSBA-15, and xFe-ZrSBA-15, where x = theoretical wt% Fe loading.

### 3.2. Characterization

The prepared catalysts were characterized by X-ray diffraction (XRD), transmission electron microscopy (TEM), N_2_ physisorption, temperature-programmed reduction (TPR-TGA), and XPS to determine their physiochemical properties. The X-ray Powder Diffraction (XRD) patterns were obtained using a Siemens D5000 using CuKα radiation (l = 1.5406 Å). The samples were scanned over a range of 0.5–80° 2*θ* with steps of 0.04°. The N_2_-physisorption isotherms were measured on a Tristar 3000 apparatus (Micromeritics). The samples were outgassed at 473 K for 2 h in the port of the adsorption analyzer. The BET-specific surface area was calculated from the relative pressure range from 0.05 to 0.21 [25]. The total pore volume was estimated on the basis of the amount adsorbed at a relative pressure of 0.96. The pore size distributions (PSDs) were calculated from nitrogen adsorption data using an algorithm based on the ideas of Barrett, Joyner, and Halenda (BJH) [26]. Scanning electron microscopy (SEM) images and EDAX data were obtained using a high-resolution scanning electron microscope Zeiss Supra TM 3VP equipped with an energy-dispersive X-ray spectrometer INCA 400 (OXFORD INSTRUMENTS 7659, Abingdon, UK).

The X-ray photoelectron spectroscopy (XPS) measurements were carried out using an AXIS Supra electron-spectrometer (Kratos Analytical Ltd.), Manchester M17 1GP, UK) fitted with a monochromatic AlKα radiation with a photon energy of 1486.6 eV. The energy calibration was performed by normalizing the C1s line of adsorbed adventitious hydrocarbons to 284.6 eV. The binding energies (BE) were determined with an accuracy of ±0.1 eV. The chemical composition of the samples was determined by monitoring the areas and binding energies of O1s, Si2p, Al2p, Fe2p, Fe3p, Zr3d, and S2p photoelectron peaks. Using the commercial data-processing software of Kratos Analytical Ltd. the concentrations of the different chemical elements (in atomic %) were calculated by normalizing the areas of the photoelectron peaks to their relative sensitivity factors.

The temperature-programmed reduction-thermogravimetric analysis (TPR-TGA) was performed using a STA449F5 Jupiter-type instrument of NETZSCH Gerätebau GmbH, Netzsch, Germany. About 20 mg of sample was placed in a microbalance crucible and heated in a flow of 5 vol. % H_2_ in Ar (100 cm^3^/min) up to 600 °C at 5 °C/min and held for 1 h. Prior to the TPR experiments, the samples were treated in situ in an airflow (10 °C/min) up to 400 °C, followed by a hold-up of 1 h to remove physisorbed water.

### 3.3. Catalytic Activity Measurements

Pretreatment of the catalysts was carried out in air at 450 °C for 1 h for removal of moisture and physisorbed gases. The reaction of toluene oxidation was monitored at atmospheric pressure using a vertical fixed-bed flow reactor with air as carrier gas (30 mL/min). In a typical reaction, 30 mg of catalyst with particle size 0.2–0.8 mm mixed with 60 mg glass beads (inactive) of the same diameter was tested. The sample was placed in a bed in the middle of a quartz tube of a 15 mm inner diameter reactor. To assure precise measurement of the catalyst temperature, a thermocouple was positioned in the catalyst bed. The temperature of all gas lines of the apparatus was kept constant at 110 °C to avoid adsorption of the monitored gas on the tube walls. The airflow was purged through toluene equilibrated at 0 °C (p_toluene_ = 0.9 kPa). The reactor was fed with a flow rate of 30 mL/min and catalytic tests were carried out in the temperature range of 120–550 °C at a weight hour space velocity (WHSV) of 1.2/h. The results for each temperature were recorded after reaching of reaction steady state (30 min). Online analysis of the reaction products was followed using a NEXIS GC-2030 ATF (Shimadzu, Japan) with a 25 m PLOT Q capillary column. The turnover frequency (TOF) was calculated as the converted number of toluene molecules per metal atom per second.

## 4. Conclusions

Iron-modified SBA-15, AlSBA-15, and ZrSBA-15 materials were successfully prepared. The formation of finely dispersed iron oxide species can be assumed based on the XRD, XPS, and TPR data. It was found that the surface properties of the mesoporous support due to the presence of Al or Zr determined the state of the formed iron oxide species, their dispersion, and reducibility. The obtained Fe-containing SBA-15 and AlSBA-15 samples showed high catalytic activity in toluene oxidation at higher temperatures (280–450 °C). However, the 5 wt. % Fe-containing ZrSBA-15 catalyst showed excellent catalytic activity at lower temperatures (150–380 °C) most probably due to the synergistic effect of Fe-Zr leading to the presence of accessible and stable Fe^2+^/Fe^3+^ redox active sites. This main finding will be in the focus of our further investigations on Fe/Zr-containing systems.

## Figures and Tables

**Table 1:** Elemental composition, surface area, and pore characteristics of the materials.

Sample\Element	Fe (wt.%)	Al (wt.%)	Zr (wt.%)	S_BET_ (m^2^/g)	V_tot_ (cm^3^/g)	W_BJH_ (nm)
SBA-15	-	-	-	563	0.833	7.1
0.5Fe-SBA-15	0.47	-	-	361	0.452	7.0
1Fe-SBA-15	1.02	-	-	366	0.482	7.1
2Fe-SBA-15	2.11	-	-	352	0.458	7.2
5Fe-SBA-15	4.86	-	-	400	0.479	6.8
Al-SBA-15	-	1.43	-	712	1.011	8.7
0.5Fe-AlSBA-15	0.41	1.57	-	447	0.600	8.8
1Fe-AlSBA-15	1.00	1.64	-	415	0.542	8.9
2Fe-AlSBA-15	2.11	1.38	-	395	0.504	8.8
5Fe-AlSBA-15	4.77	1.29	-	422	0.482	8.4
Zr-SBA-15	-	-	4.77	759	0.891	7.9
0.5Fe-ZrSBA-15	0.48	-	4.25	378	0.427	7.9
1Fe-ZrSBA-15	1.05	-	4.84	363	0.414	7.8
2Fe-ZrSBA-15	2.07	-	4.58	382	0.411	7.7
5Fe-ZrSBA-15	4.94	-	4.47	467	0.452	7.7

Vtot = total pore volume evaluated from adsorption isotherm at the relative pressure of 0.96; S_BET_ = BET surface area; W_BJH_ = mesopore diameters at the maximum of the BJH pore size distribution.

**Table 2:** Surface chemical composition of 5Fe-SBA-15, 5Fe-AlSBA-15, and 5Fe-ZrSBA-15.

Samples	O (at.%)	Si (at.%)	Fe (at.%)	Zr (at.%)	Al (at.%)
5Fe-ZrSBA-15	61.8	36.5	0.8	0.9	-
Spent 5Fe-ZrSBA-15	64.4	34.1	0.7	0.8	-
5Fe-SBA-15	63.1	36.5	0.4	-	-
5Fe-AlSBA-15	63.6	35.3	0.3	-	0.8

## Data Availability

Not available.

## Supplementary Materials

The following supporting information can be downloaded at: https://www.mdpi.com/article/10.3390/molecules28010204/s1. Figure S1. XRD patterns of iron modified SBA-15 (A), AlSBA-15 (B) and Zr-SBA-15 (C) samples. Figure S2. Zr3d XPS spectra of initial and spent 5Fe-ZrSBA-15 sample.

## Sample Availability

Samples of the iron-modified SBA-15, AlSBA-15, and ZrSBA-15 materials are available from the authors.
