# Supplementary material for: Low-Temperature Toluene Oxidation on Fe-Containing Modified SBA-15 Materials
Source: Molecules. 2022 Dec 26;28(1):204. doi: 10.3390/molecules28010204 (PMC9821885; doi:10.3390/molecules28010204)
Supplement: Supplementary file 1 [file molecules-28-00204-s001.zip › molecules-2082720-supplementary.pdf]

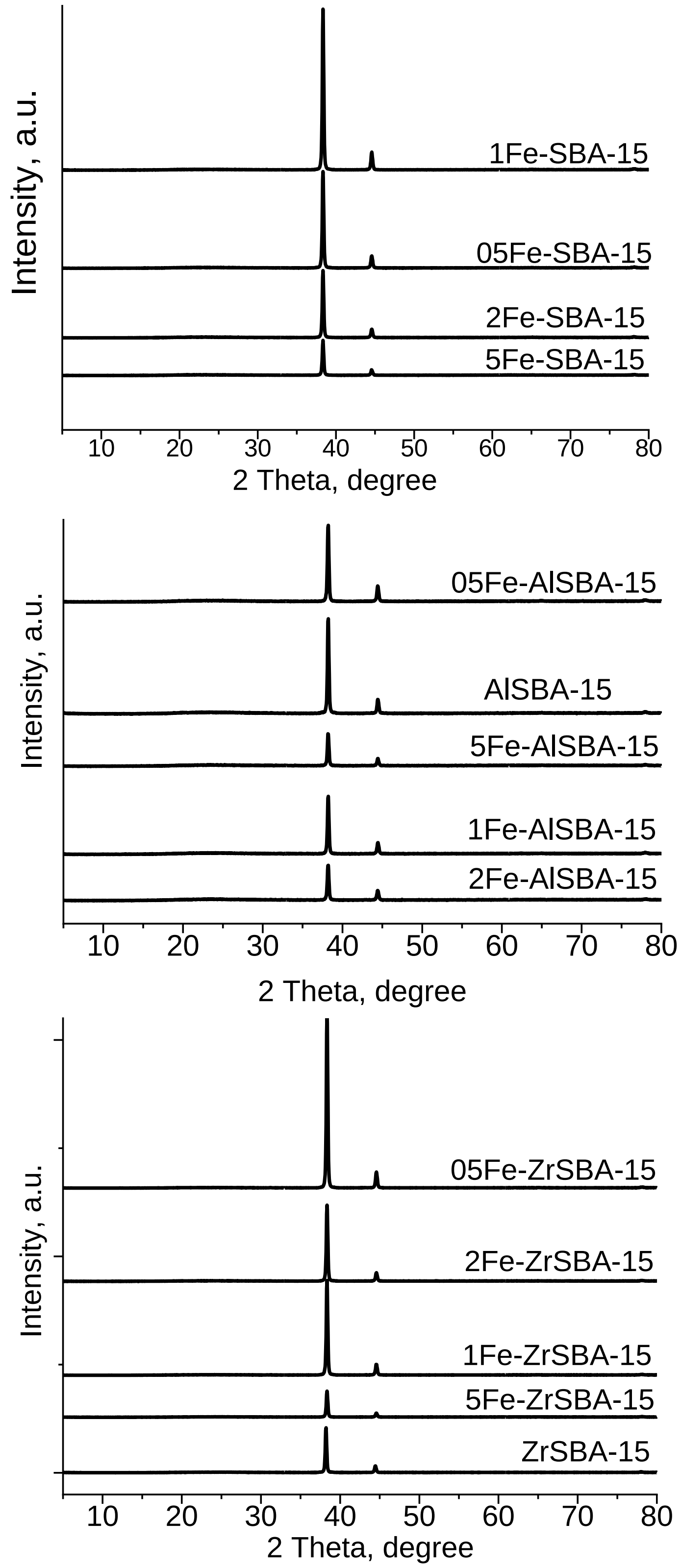

Figure S1. Figure S1. XRD patterns of iron modified SBA-15 (A), AlSBA-15 (B) and Zr-SBA-15 (C) samples.

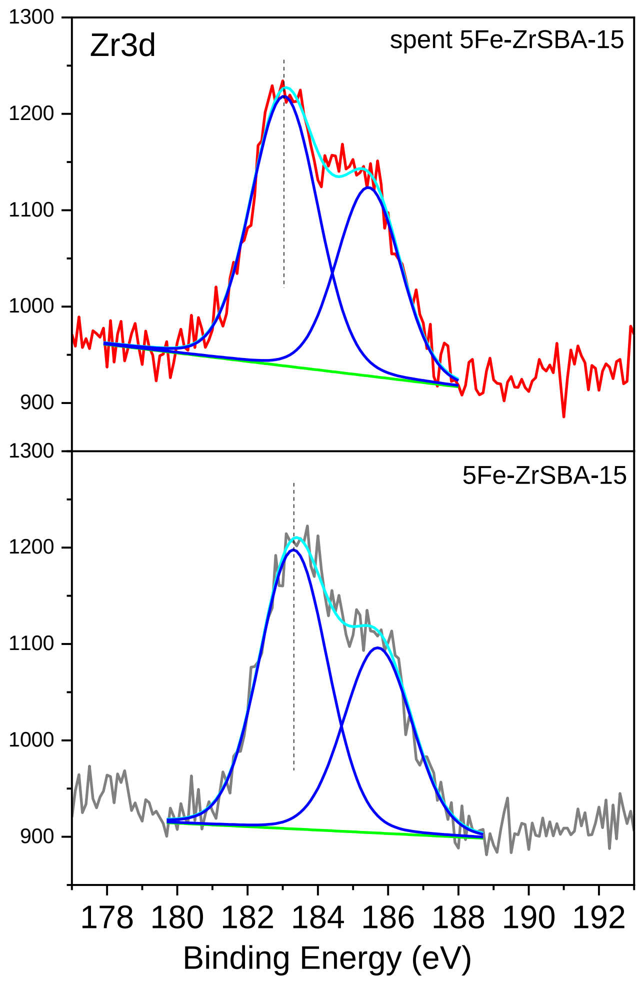

Figure S2. Zr3d XPS spectra of initial and spent 5Fe-ZrSBA-15 sample.
